# Primary spinal Non-Hodgkin Lymphoma presenting as impending cauda equina syndrome: A case report

**DOI:** 10.1016/j.amsu.2022.104696

**Published:** 2022-09-15

**Authors:** Prashant Adhikari, Sulochana Khadka, Pradeep Raj Regmi, Anjan Shrestha, Bhaskar Raj Panta, Sandeep Bhandari, Emre Acaroglu

**Affiliations:** a HAMS Hospital, Kathmandu, Nepal; b Nepalese Army Institute of Health Sciences, College of Medicine, Kathmandu, Nepal; c Tribhuvan University Teaching Hospital, Kathmandu, Nepal; d Ankara Spine Center, Turkey

**Keywords:** Case report, Decompression, Primary spinal lymphoma, Non-hodgkin lymphoma, Diffuse large B cell Lymphoma

## Abstract

**Introduction:**

Malignant lymphoma (ML) can involve the central nervous system either primarily or by secondary spread, which tends to occur late in the disease as part of widespread dissemination. Lymphoma presenting as primary tumors of the spinal cord is extremely uncommon. Primary spinal lymphoma if detected early can have a good prognosis with no relapse after effective treatment.

**Case presentation:**

A 32 years old male patient presented with the symptoms of impending cauda equina syndrome which was managed with surgery and chemotherapy. The patient was successfully treated without the relapse of his condition at his 6 months follow-up scan.

Discussion: Primary spinal non-Hodgkin lymphoma is a rare entity among extranodal non-Hodgkin lymphoma. MRI is usually non-confirmatory and needs immunohistochemistry for the correct diagnosis. R–CHOP regimen is the standard chemotherapy regimen. Surgical decompression is required in cases of impending neurological injury along with radiotherapy.

**Conclusion:**

Primary spinal epidural diffuse large B‐cell lymphoma should be considered as a differential diagnosis in patients presenting with back pain and symptoms of impending cauda equina syndrome. It is important to early detect and treat the disease to prevent permanent neurological injury and metastasis.

## Highlights

• Primary spinal epidural diffuse large B‐cell lymphoma should be considered as a differential diagnosis in patients presenting with back pain and symptoms of impending cauda equine syndrome.
• Surgical decompression may be required in cases of impending neurological injury.
• Early diagnosis and treatment is the key to have a better prognosis.

## 1 Introduction

Primary spinal lymphoma is an entity of lymphomas that involves the vertebra as well as the spinal cord along with adjacent paravertebral soft tissues without any recognizable other sites of lymphomas at diagnosis. A spinal location for lymphoma is observed in 0.1–6.5% of all lymphomas which is a rare condition [1]. Thus, it is challenging to diagnose and may easily be misdiagnosed. Extranodal non-Hodgkin's lymphoma (NHL) accounts for 24–48% of all NHL, while primary spinal epidural lymphoma comprises 0.9% of all extranodal NHLS [2]. Spinal cord compression is an uncommon primary manifestation and requires to be treated with surgery for the purpose of diagnosis and decompression [3]. The diagnosis is challenging due to its atypical clinical presentation and the difficulty in establishing a conclusive tissue diagnosis with MRI and core biopsy [4]. Non-Hodgkin's lymphoma with spinal epidural involvement at presentation is an aggressive disease because of which intensive treatment combining irradiation with chemotherapy, and surgery as needed, which is usually suggested in order to achieve good local response and long-term survival [5]. Here, we present a 32 years old male with the diagnosis of primary diffuse large B Cell Lymphoma of the spine treated successfully with decompression surgery and chemotherapy. This case report has been written as per SCARE 2020 criteria [[6], [6]].

## 2 Case Presentation

A 32 years old male initially presented to another center with complaints of pain in the lumbar region for the duration of 6 months. The pain was insidious in onset, gradually progressive radiating to the bilateral lower limbs. There was no associated fever, weight loss, chest pain, cough, shortness of breath, or loss of consciousness. He had no significant past history. There was no history of similar illnesses in the family. He was a nonsmoker and non-alcoholic.

**Fig. 1:**
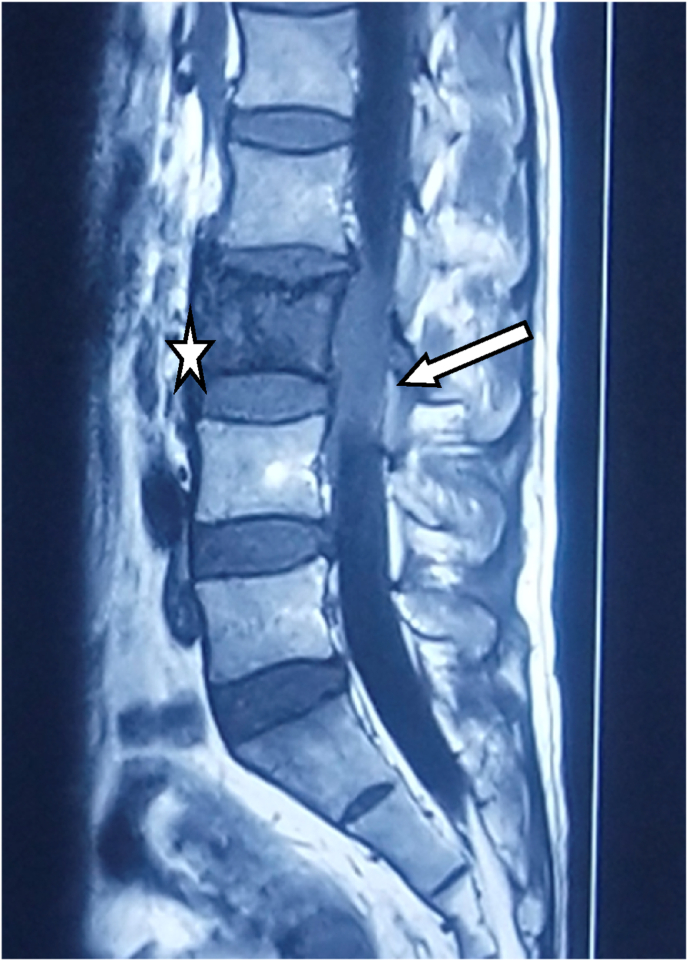
MRI shows T1 intermediate signal intensity lesion in the epidural region (white arrow) of L3 vertebral body along with diffuse low signal intensities within the vertebral body (asterisk). Height of the vertebra is maintained.

On further investigation, MRI was done which revealed a T1 intermediate signal intensity lesion in the epidural region of the L3 vertebral body along with diffuse low signal intensities within the vertebral body (Fig. 1). A biopsy was done which showed inflammatory cells. The initial differential diagnosis as tuberculosis was made. Hence, empiric anti-tubercular therapy was started for him. However, after 2 months, his symptoms progressed rapidly with difficulty in the bladder and bowel function, night cries, saddle anaesthesia, and referred pain in bilateral lower extremities because of which he sought our medical advice. On examination, there was a sluggish ankle (2/5) and knee reflex (2/5) bilaterally. He was found to have difficulty walking.

**Fig. 2:**
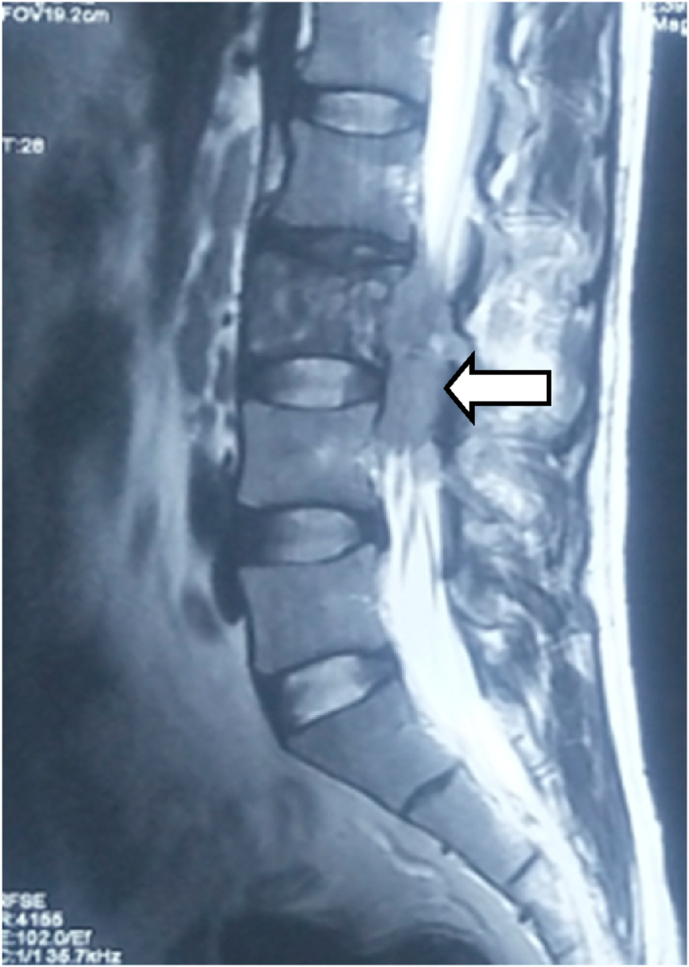
Second MRI done after 2 months shows similar T2 intermediate signal intensity soft tissue lesion in the epidural location (white arrow) of L3 vertebra with diffuse low signal intensity within the vertebral body.

**Fig. 3:**
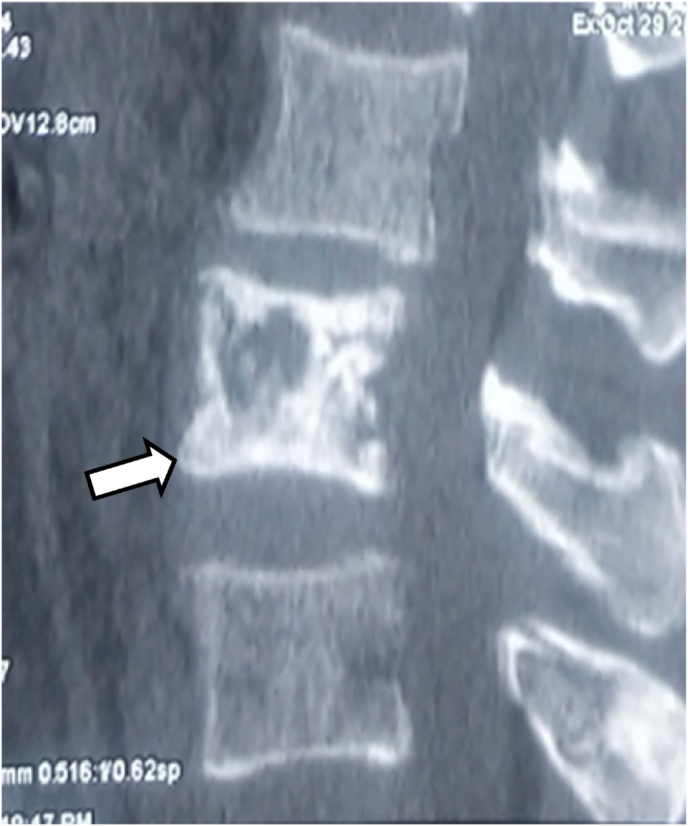
Sagittal CT shows diffuse sclerosis (white arrow) of the involved vertebra with ill-defined lytic areas.

**Fig. 4:**
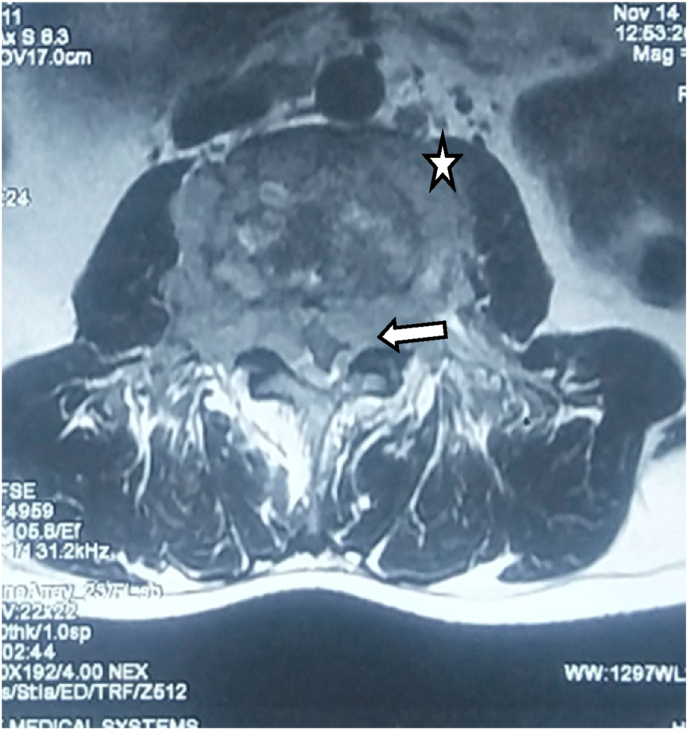
MRI axial T2 image shows circumferential epidural soft tissue lesion (white arrow) causing significant central spinal canal stenosis; Prevertebral as well as paravertebral soft tissue components are also seen (asterisk).

Contrast MRI was done which showed T2 low signal intensity lesions within the L3 vertebral body including the posterior elements along with circumferential soft tissue density showing T2 intermediate to high signal intensity soft tissue density in the epidural region causing severe narrowing of the central spinal canal and bilateral lateral recesses at L3 level (Fig. 2, Fig. 3, Fig. 4). L2-L3 disc showed low signal intensity. There was STIR high signal intensity in the prevertebral as well as bilateral paravertebral regions. These findings were not typical for tuberculosis which is usually common in our setup. Other investigations like Chest X‐ray, complete blood count (CBC), liver and renal function tests, erythrocyte sedimentation rate (ESR), c‐reactive protein (CRP), peripheral blood smear, lactate dehydrogenase (LDH), uric acid, and urinalysis were normal.

**Fig. 5:**
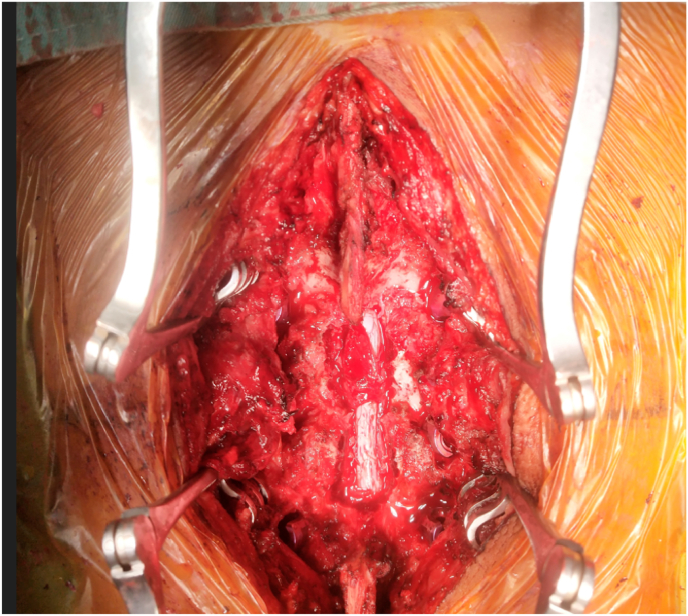
Soft tissue attachment at the dura mater.

**Fig. 6:**
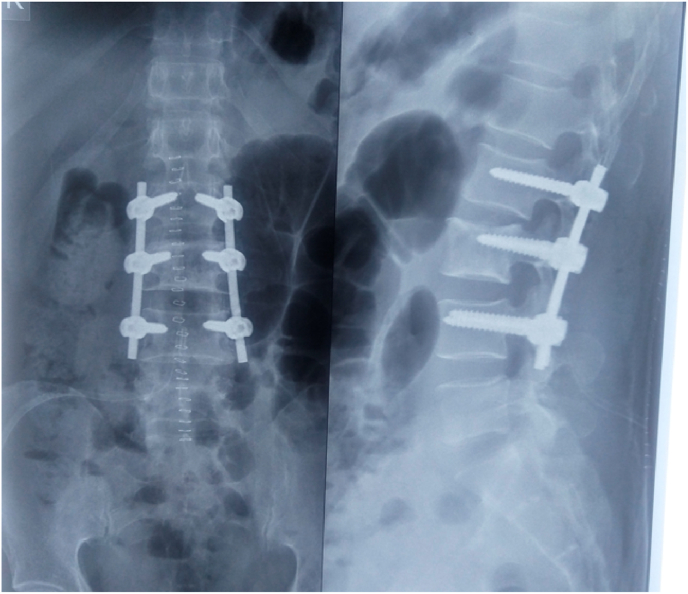
Posterior decompression and stabilization (L2-L4).

Since he had symptoms of spinal cord compression, the next step of management was decompression. During the procedure, the soft tissues adhering around the dura mater were visible (Fig. 5). Following decompression, posterior instrumentation was performed (Fig. 6). The excised specimen was taken for gram stain, gene Xpert and histopathological evaluation.

The gram stain and gene Xpert report did not show any significant findings. Finally, histopathological features revealed chronic osteomyelitis with focal lymphoid cell proliferation. The differential diagnosis at this time was osteomyelitis. However, the Non-Hodgkin Lymphoma panel was suggested to rule out lymphoma. The immunohistochemistry report finally revealed diffuse large B cell Lymphoma. The immunomarkers like CD20, Ki 67, BLC 2, MUM-1, CD-10, and BCL 6 were reactive. CT chest, neck, and abdomen were performed which showed no signs of lymphadenopathy. There was no other primary site hence the finding of primary lymphoma of the spine was made. He was started with the chemotherapy regimen R–CHOP (rituximab, cyclophosphamide, hydroxydaunorubicin hydrochloride (doxorubicin hydrochloride), vincristine (Oncovin), and prednisone). A total of 6 cycles of the treatment were provided every 21 days for the duration of five months. Subsequently, he completed the cycles of chemotherapy. Post procedural CT scan of chest, neck and abdomen after 6 months showed no signs of recurrence or metastasis.

Currently, the patient is asymptomatic and free of disease. He is performing his regular activities.

## 3 Discussion

Epidural lymphomas form 9% of all spinal epidural tumors [1]. It is debated whether the primary spinal epidural lymphoma (PSEL) has originated from lymphoid tissue in the epidural space or from either paraspinal, spinal, or retroperitoneal tissues, accessing the epidural space via the interspinal foramina [[6], [6],^7^]. The most common site for its location is the lumbar or lower dorsal area [8]. Thoracic spine location is a significant poor prognostic factor [9]. In our case, the patient presented with lumbar pain and was found to have a lesion in the lumbar (L3) vertebrae. The most common age of presentation is in the fifth to seventh decade of life with more than 80% being older than 40 years [1]. However, our patient was a young man in his mid-30s. Spinal cord compression may occur in 0.1%–10.2% of NHL patients during the course of the disease and is usually aggressive in behavior [10]. In our patient, there were symptoms of impending spinal cord compression which was managed with decompression. After surgery, he had improvement in his symptoms, and excised tissue was sent for histopathological and immune-histochemical examination subsequently.

Because bony involvement is not present, plain radiographs rarely provide clinically useful information. Myelography and computerized tomography (CT)-myelography, as well as magnetic resonance imaging (MRI), are useful for detecting epidural compression [11]. MRI appearance of PSEL in our patient was isointense on T1-weighted images and iso to hyperintense on T2-weighted images, with marked contrast enhancement. The signal was homogeneous in all the sequences. This is similar to the description by Negendank et al. of lymphomas elsewhere in the body and the report by Li et al. regarding spinal epidural lymphomas. There does not appear to be a change with differing grades of lymphomas [12,13].

Most primary spinal lymphomas are comprised of diffuse large B-cell lymphomas with a minority being follicular lymphoma, precursor B-lymphoblastic lymphoma, Ki-lymphoma, diffuse lymphoblastic lymphoma, small lymphocytic lymphoma, or T-cell lymphoma [13]. In our patient as well, diffuse large B cell lymphoma was confirmed through immunohistochemistry. It was positive for CD20, Ki 67, BLC 2, MUM-1, CD-10, and BCL 6. Surgical decompression is recommended in patients with signs of spinal cord injury in order to prevent irreversible neurological damage and is related to high rates of disease-free survival [14]. In our patient, as soon as the symptoms of impending cauda equine were evident, immediate surgical decompression was performed.

All patients with a histological diagnosis of lymphoma need a complete systemic workup for lymphoma, without which a diagnosis of PSEL cannot be made. In the series by Monnard et al., whole-body CT scan, bone marrow assessment, cerebrospinal fluid examination, lactic dehydrogenase, and white blood cell count were measured [15]. In our patient, whole-body CT scan along with blood investigations were performed to examine the presence of lesions in other parts of the body. Based on the current practices, well-established treatments, such as radiotherapy and chemotherapy or a combination of both should remain the mainstays and surgery should be used in combination with radiotherapy and/or chemotherapy [16]. Most studies have suggested that combined modalities of treament including radiotherapy and chemotherapy seem to be effective treatments [15]. Modified CHOP chemotherapy with rituximab + CHOP (R–CHOP) is the most commonly used chemotherapy regimen [17]. In our patient, chemotherapy with (R–CHOP) was the mainstay of treatment of his disease. However, he was not treated with radiotherapy.

A study by Lyons et al. has concluded that primary spinal epidural non-Hodgkin's lymphoma can be associated with a favorable outcome if diagnosed and treated early [[6], [6]]. It's prognosis is also better than the prognosis of nodal non-Hodgkin lymphoma [18]. It is similar in line of our case report where the patient had complete recovery after the chemotherapy and his follow-up whole-body CT scan did not reveal any signs of relapse.

## 4 Conclusion

Primary spinal epidural diffuse large B‐cell lymphoma should be considered as a differential diagnosis in patients presenting with back pain and symptoms of impending cauda equine syndrome. Early diagnosis and treatment are the keys to having a better prognosis. Surgical decompression may be required in cases of impending neurological injury.

## Ethical approval

N/A.

## Sources of funding

None.

## Author contributions

Author 1: concept of study, contributed in writing the case information.

Author 2: Literature review, writing initial draft, revising, and editing the manuscript.

Author 3: Literature review and writing case information.

Author 4: Literature review, revising and editing the manuscript.

Author 5: Literature review and editing the manuscript.

Author 6: Literature review and editing the manuscript.

Author 7: Literature review and revising the manuscript.

All authors were involved in manuscript drafting and revising, and approved the final version.

## Registration of research studies

1. Name of the registry: N/A.
2. Unique Identifying number or registration ID: N/A.
3. Hyperlink to your specific registration (must be publicly accessible and will be checked): N/A.

## Consent

Written informed consent was obtained from the patient for publication of this case report and accompanying images. A copy of the written consent is available for review by the Editor-in-Chief of this journal on request.

## Guarantor

Sulochana Khadka, Nepalese Army Institute of Health Sciences – College of Medicine, Kathmandu, Nepal. Email: suloachankhadka1@gmail.com, Phone: +977–9848559942.

## Provenance and peer review

Not commissioned, externally peer-reviewed.

## Declaration of competing interest

None.
